# Determination of selected chemical parameters in surface water samples collected from the Revelva catchment (Hornsund fjord, Svalbard)

**DOI:** 10.1007/s00706-016-1771-1

**Published:** 2016-06-07

**Authors:** Klaudia Kosek, Żaneta Polkowska

**Affiliations:** Department of Analytical Chemistry, Faculty of Chemistry, Gdansk University of Technology, 11/12 Narutowicza St., 80-233 Gdańsk, Poland

**Keywords:** Arctic environment, Climate change, Data base, Ecology, Long-range transport, Spectrophotometry

## Abstract

**Abstract:**

Surface water samples (river and lake) were collected from the Revelva catchment every summer from 2010 to 2013. This study concerns importance of the use of some analytical techniques for pollutants and parameters determination in Arctic environmental samples based on the example of total organic carbon, phenols, and formaldehyde determination and measurement of pH and electrical conductivity parameters. Significant average concentration levels of formaldehyde were observed in 2012 and reached 0.26 mg/dm^3^. Furthermore, the highest determined levels of total organic carbon and electrical conductivity were observed in samples collected in summer 2013. The average value of total organic carbon in that year was 9.54 mg/dm^3^, and electrical conductivity increased to 63.0 µS/cm. The results of surface water samples analyses show an increasing trend in pollutants concentration levels over the years what may suggest that each year the emission of contaminants from lower latitudes is bigger and, consequently, more of them are deposited in the Arctic.

**Graphical Abstract:**

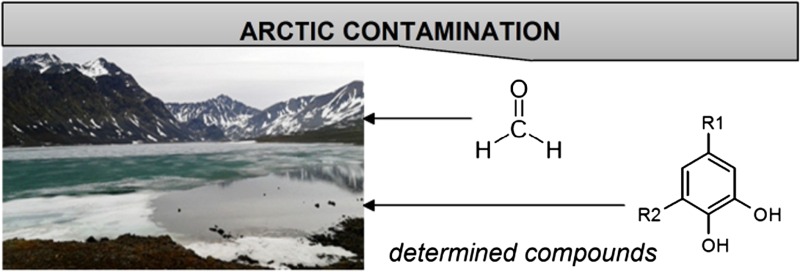

## Introduction

The Arctic encompasses the lands and north waters, including approximately 20 million km^2^ of ocean, all of Iceland and Greenland, the northern reaches of Norway, Finland, Russia, Canada north of the southern shore of Hudson Bay, and Alaska north of the panhandle in the United States [1].

The Svalbard archipelago is located at the NW limit of the European continental shelf between 76.50°–80.80°N and 10°–34°E [2]. It is one of the tenth most glaciated areas in the Arctic. The total area of Svalbard is 62,800 km^2^, and glaciers cover about 60 % of the land [3]. There are many good research publications, review articles, and summaries where the Arctic region has been described [4–8]. During the past decade, the Arctic has undergone dramatic change, and it is not consider a pristine region any more. An interest in the processes of transport of a wide range of pollutants and them fate in the polar regions located distantly from industrial centers is still increasing. The current analytical techniques enabling conducting studies prove that the Arctic has become an area of highly intensive anthropopressure, and it is possible to recognize considerably low, but still harmful and persisting levels of pollutants concentration present there [4, 5].

Chemical substances of anthropogenic origin that appear in the polar regions are often considered as persistent organic pollutants [POPs, e.g., polycyclic aromatic hydrocarbons (PAHs), polychlorinated biphenyls (PCBs), dichlorodiphenyltrichloroethanes (DDTs)], metals, phenols, formaldehyde, and radioactive isotopes. Accumulation of these compounds in northern latitudes is a well-documented phenomenon [4, 5, 8], and it is thought to occur for a variety of reasons. In a process known as a global distillation, prevailing ocean and wind currents bring contaminants to the Arctic where they are subsequently trapped in all kinds of environmental reservoirs by the cold climate. This process is often called the grasshopper effect, as chemicals repeatedly evaporate and condense as long as they reach the Arctic. In addition to the long-range transport of pollutants, contaminants appearing in Svalbard are also the consequence of some small local sources, such as military installations, industrial outlets, settlements, and ships [9].

The studies described in the article mainly focus on the pollution caused by the appearance of phenolic compounds and formaldehyde in environmental samples. Parameters, such as total organic carbon, electrical conductivity, and pH, have been added additionally. This kind of information may complete the knowledge of the contaminants (principally persistent organic pollutants) present in the polar regions, including the Arctic. This may also be helpful in identifying sources of pollution inputs to the Arctic.

## Results and discussion

**Fig. 1:**
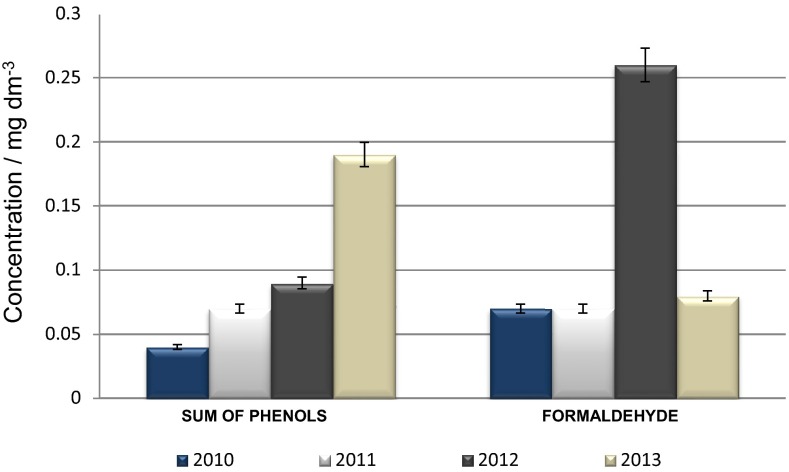
Average values of sum of phenols and formaldehyde concentration levels in samples collected every summer from 2010 to 2013 from Revelva catchment

Total organic carbon (TOC) is the amount of carbon bound in an organic compound, and it is the sum of suspended and dissolved organic carbon. This constitutes a huge range of compounds with a variety of properties. TOC is released to environment from natural (metabolism, excretion, and deposition of organisms) and anthropogenic (sewage treatment plants, farm slurry, and silage runoff) sources. As well as TOC, in polar environmental samples, formaldehyde and sum of phenols may be found. They constitute a significant amount of pollutants transported to the Arctic from lower latitudes [10]. Figure 1 shows the average values of sum of phenols and formaldehyde concentration levels observed in collected samples from summer 2010 to 2013.

Significantly high concentration levels of formaldehyde in 2012 may be caused due to increased emission of pollutants from industrialized areas compared with other years and its transport to the Arctic. Moreover, eruptions of two main volcanoes in Iceland: Eyjafjallajökull volcano (63.63°N, 19.62°W) and Grímsvötn volcano (64.42°N, 17.33°W), may have had an impact on the results as well. Regarding to sum of phenols concentration levels in 2012, there are not significant occurrence of them that year comparing to formaldehyde content. Volcanic eruptions are not the main source of phenolic compounds, and they are not transported to the Arctic in such high concentrations. Phenolic compounds are more likely to be dissolved within the pore water than transported on the surface of the ash particles. In conclusion, compounds from phenolic group are more likely to partition to water than air. Despite this, it can be observed an increasing trend in sum of phenols concentration levels over 4 years that may be an important indicator of climate change.

**Fig. 2:**
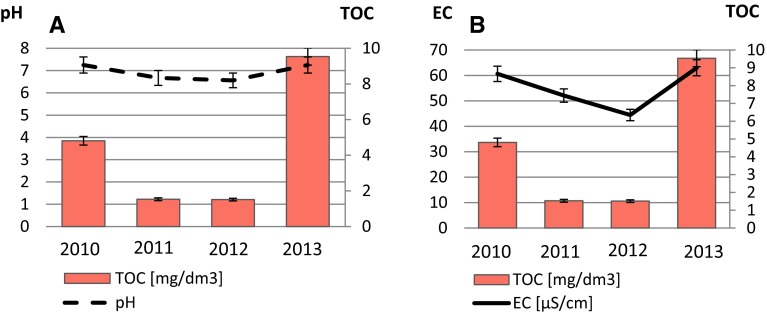
Average values of pH, electrical conductivity, and total organic carbon in analyzed samples

The hydrochemical studies of polar areas were carried out over many years in the surroundings of Polish Polar Station at Hornsund, also in the catchment of Revelva, and demonstrated high hydrochemical variability. In these studies, it may be observed that surface water samples were characterized by slightly acidic pH (Fig. 2a).

The highest determined levels of total organic carbon and electrical conductivity were observed in samples collected in summer 2013. The average value of total organic carbon in that year was 9.54 mg/dm^3^, and electrical conductivity increased to 63.0 µS/cm. These high levels of TOC concentration might be caused due to appearance of some industrial catastrophes that occurred in 2013 and were not associated with volcanic eruptions. The lowest average values of these parameters were observed in two previous years. Summing up, TOC and electrical conductivity parameter are substantially related: higher conductivity reflects higher sample pollution (Fig. 2b). However, higher level of EC may also suggest the presence of other chemical compounds in analyzed samples that do not contain organic carbon.

**Fig. 3:**
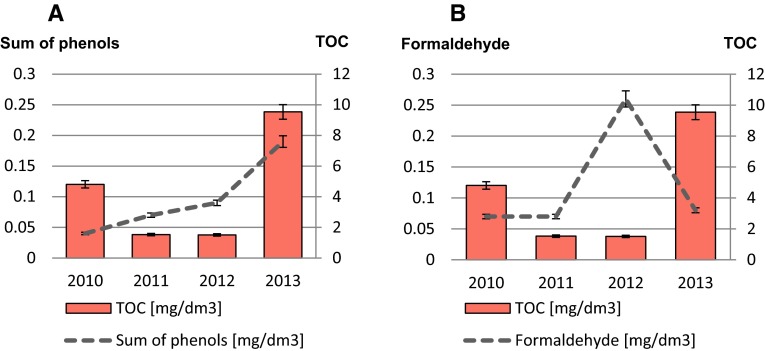
Average values of sum of phenols, formaldehyde, and total organic carbon in analyzed samples

Figure 3 shows correlations between sum of phenols, formaldehyde, and total organic carbon. The correlation between TOC and sum of phenols is visible on the graph above (Fig. 3a). It may be stated that with the increase in sum of phenols concentrations, total organic carbon concentration levels also increase. Another situation is presented on the next graph (Fig. 3b). Even though the concentration levels of TOC are still higher than formaldehyde content, the correlation between these compounds is altered. For instance, in summer 2013, concentration levels of TOC reached almost 10 mg/dm^3^ and formaldehyde content in surface water samples were practically insignificant in that year. It can be explained by the occurrence of other carbon compounds in the Arctic, such as black carbon (BC), which emitted into the atmosphere, warms the Arctic in 2 days or methane. To describe correlation between TOC and phenolic compounds concentration levels and TOC and formaldehyde content, the mathematical formulas have been created on the basis of the linear function *y* = *ax* + *b*. In addition, to confirm presented data relationships, Pearson’s correlation coefficients (*r*) have been added to measure the strength of a linear correlation between paired data (Table 1).

**Table 1:** Summary of the correlation results of sum of phenols and formaldehyde concentration levels related to the TOC content [11]

Relationship	*a*	*b*	*r*	The intensity of correlation
TOC/sum of phenols	0.013	0.042	+0.75	Strong correlation
TOC/formaldehyde	0.011	0.169	−0.46	Moderate correlation

Scale of correlation intensity: *r* = 0.00–±0.19: very weak, *r* = ±(0.20–0.39): weak, *r* = ±(0.40–0.59): moderate, *r* = ±(0.2 ± (0.60–0.79)): strong, *r* = ±(0.80–1.0): very strong

Water reservoirs contain carbon in various forms. In the ocean, dissolved organic carbon comes from decaying biological material. Dissolved inorganic carbon includes carbon dioxide and other simple molecules and ions containing carbon. Both organic and inorganic are also present in particulates. Lakes and rivers are responsible for most of the direct transport of carbon from land to ocean. The Arctic holds 36 % of the world’s lake surface area and accounts for 10 % of global river discharge to the ocean. Taking the same proportions of estimates of global freshwater carbon releases gives an estimate for the Arctic of 25–54 million tonnes of carbon from lakes each year and 15–30 million tonnes from rivers. Transport of carbon in the Arctic is important for determining where it may be emitted to the atmosphere or captured in all kinds of environmental elements. There is considerable uncertainty involved in most estimates of carbon transport and its emission, but river transport and ocean currents appear responsible for the largest amounts [12].

## Conclusion

According to actual scientific knowledge, a wide range of pollutants are transported and distributed throughout the Arctic through natural distribution pathways, including the atmosphere, transpolar ice drift, ocean currents, and rivers [13]. The results of analyses of surface water samples from the Revelva catchment show an increasing trend in pollutants concentration levels over the years. The highest concentration levels of sum of phenols were observed in samples collected in summer 2013 and reached 0.19 mg/dm^3^, and the highest concentration levels of formaldehyde were observed in those ones collected in summer 2012 and reached 0.26 mg/dm^3^ due to two large volcanic eruptions in Iceland. In addition, the highest determined levels of total organic carbon and electrical conductivity were observed in samples collected in 2013. The average value of TOC in that year was 9.54 mg/dm^3^, and electrical conductivity increased to 63.0 µS/cm. Obtained results may suggests that each year the emission of contaminants from lower latitudes is bigger, and more of them are deposited in the Arctic what may serve as early warning signal of expected climate change. During the past few decades, monitoring and fate studies of pollutants in the Arctic have been conducted mainly in the marine environment and the surrounding coastal zones. Only a few comprehensive studies exist on the fate and distribution of pollutants in the terrestrial environment and freshwater reservoirs [13]. Therefore, it is important to continue monitoring contaminants concentration levels of whole area and to develop current analytical techniques for all kinds of xenobiotics determination, because even low pollutants concentration levels in the polar regions may suggest a significant contamination of studied area.

## Experimental

### Study area

**Fig. 4:**
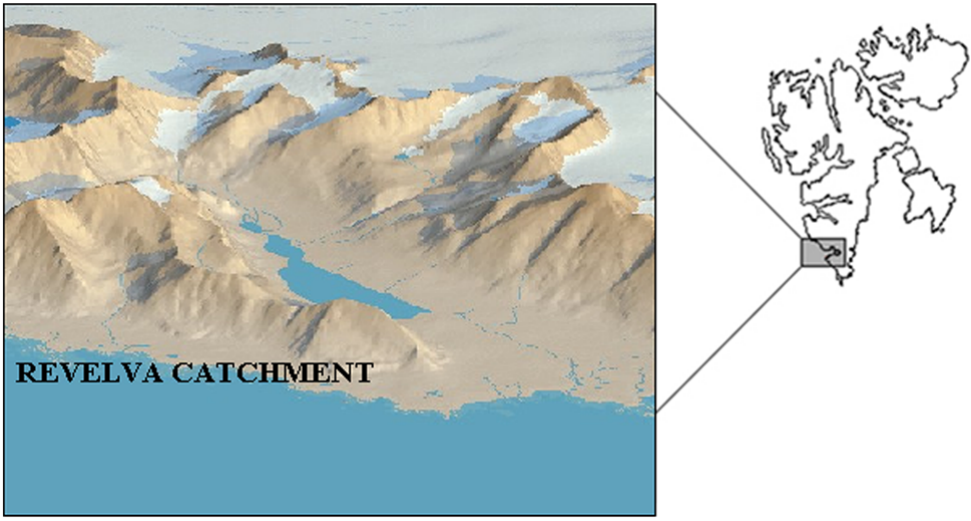
Map of Svalbard considering location of the Revelva catchment (based on the toposvalbard.npolar.no)

The study area covered the southern part of Wedel-Jarlsberg Land-Spitsbergen (Svalbard archipelago). The primary study object was the Revelva catchment in the vicinity of Polish Polar Station at Hornsund (Fig. 4).

The main river (Revelva) is fed both directly by atmospheric precipitation, snow melt water streams, and a river originating from the Arie glacier. Revelva drains into the bay of Ariebukta in the south, forming an estuary. In the upper part of the catchment, the main streams originate from the slopes of Eimfjellet (640 m asl) and Skålfjellet (635 m asl) [10]. The Revelva catchment has only one small glacierized part, but past glacial activity has left traces in its upper part. The bottom part of the Revelva valley is an elevated marine terrace, with abrasion stacks. On the terrace, areas of patterned ground and contemporary storm ridges have been formed. The diversity of the catchment landscape provides an ideal setting for a comprehensive study of processes of pollutants deposition in different parts of the abiotic environment [8].

### Sampling

Surface water samples were collected from the Revelva catchment (9 water samples collected from the Revatnet Lake and 1 sample collected from the Revelva River) every summer from July 2010 to July 2013 in the vicinity of the Polish Polar Station at Hornsund. Samples were collected in 1-dm^3^ bottles using a manual sampling technique and stored under refrigerated conditions.

### Analytical methods

The samples were transported to the laboratory of the Department of Analytical Chemistry (Gdansk University of Technology) in tightly closed bottles and stored at a temperature of 4 °C (to avoid the loss of analytes) until chemical analysis. Information on the analytical techniques used to determine total organic carbon, pH, electrical conductivity (EC), sum of phenols, and formaldehyde with their metrological characteristics is given in Table 2.

**Table 2:** Technical specifications and metrological characteristics of analytical techniques used in chemical analysis

Parameters	Measurement range	LOD^c^	LOQ^c^	Measurement equipment, method	
TOC^a^	0.150–10.0	0.030	0.100	Total organic carbon analyzer TOC–V_CSH/CSN,_ (method of catalytic combustion (oxidation) with the application of the NDIR detector)	
Electrical^b^ conductivity	–	–	–	Electrochemical method with the application of a conductometer CPC-411 by Elmetron and conductivity sensor EC 60	
pH	–	–	–	Electrochemical method with the application of a microcomputer pH by Elmetron: electrode type EPS-1	
Sum of phenols^a^	0.002–5.00	0.025	0.075	Absorbance measured at 495 nm	Spectrophotometer: Spectroquant Pharo 100 (spectrophotometric method)
Formaldehyde^a^	0.020–8.00	0.02	0.06	Absorbance measured at 585 nm	

^a^[mg/dm^3^]

^b^[µS/cm]

^c^The limit of detection (LOD) and the limit of quantitation (LOQ) were calculated based on the standard deviation of the response (s) and the slope of the calibration curve (b), according to the formulas: LOD = 3.3(s/b), LOQ = 10(s/b)

The demineralized water Milli-Q type obtained from apparatus Milli-Q^®^ Ultrapure Water Purification Systems (Millipore^®^ production) was used in chemical analysis.

## References

[1] Suk W, Avakian M, Carpenter D, Groopman J, Scammell M, Wild C (2004). Environ Health Perspect.

[2] Błaszczyk M, Jania JA, Hagen JO (2009). Pol Polar Res.

[3] Hagen JO, Kohler J, Melvold K, Winther JG (2003). Polar Res.

[4] Kozak K, Polkowska Ż, Ruman M, Kozioł K, Namieśnik J (2013). Trends Anal Chem.

[5] Ruman M, Kozak K, Lehmann S, Kozioł K, Polkowska Ż (2013). Ecol Chem Eng S.

[6] Petrov V, Morozov A, Shokalsky S, Kashubin S, Artemieva IM, Sobolev N, Petrov E, Ernst RE, Sergeev S, Smelror M (2016). Earth Sci Rev.

[7] Huang W, Lei R, Han H, Li Z (2016). Cold Reg Sci Technol.

[8] Kosek K, Lehmann S, Gajek G, Kociuba W, Franczak Ł, Polkowska Ż, Migała K, Owczarek P, Kasprzak M, Strzelecki MC (2014). Morphometric parameters of the Renardbreen as imporant factors determining the spatial distribution of chemical compounds on the glacier surface (Bellsund, Svalbard). New Perspectives in Polar Research.

[9] Wania F (2003). Environ Sci Technol.

[10] Birkenmajer K (1990). Geology of the Hornsund Area.

[11] Evans JD (1996). Straightforward statistics for the behavioral sciences.

[12] Huntington HP (2009). Update on selected climate issues of concern.

[13] Kallenborn R, Borgå K, Christensen JH, Dowdall M, Evenset A, Odland JØ, Ruus A, Aspmo Pfaffhuber K, Pawlak J, Reiersen L-O (2011). Combined effects of selected pollutants and climate change in the arctic environment.

